# Genome evolution during bread wheat formation unveiled by the distribution dynamics of SSR sequences on chromosomes using FISH

**DOI:** 10.1186/s12864-020-07364-6

**Published:** 2021-01-14

**Authors:** Yingxin Zhang, Chengming Fan, Yuhong Chen, Richard R.-C. Wang, Xiangqi Zhang, Fangpu Han, Zanmin Hu

**Affiliations:** 1 State Key Laboratory of Plant Cell and Chromosome Engineering, Institute of Genetics and Developmental Biology, Innovation Academy for Seed Design, Chinese Academy of Sciences, Beijing, 100101 China; 2 College of Agriculture, Yangtze University, Jingzhou, 434000 Hubei China; 3 United States Department of Agriculture, Agricultural Research Service, Forage and Range Research Laboratory, Utah State University, Logan, UT 84322-6300 USA; 4 College of Agriculture, University of Chinese Academy of Sciences, Beijing, 100049 China

**Keywords:** Bread wheat, Polyploidization, Simple sequence repeat, FISH

## Abstract

**Background:**

During the bread wheat speciation by polyploidization, a series of genome rearrangement and sequence recombination occurred. Simple sequence repeat (SSR) sequences, predominately located in heterochromatic regions of chromosomes, are the effective marker for tracing the genomic DNA sequence variations. However, to date the distribution dynamics of SSRs on chromosomes of bread wheat and its donors, including diploid and tetraploid *Triticum urartu*, *Aegilops speltoides*, *Aegilops tauschii*, *Triticum turgidum* ssp. *dicocoides*, reflecting the genome evolution events during bread wheat formation had not been comprehensively investigated.

**Results:**

The genome evolution was studied by comprehensively comparing the distribution patterns of (AAC)_n_, (AAG)_n_, (AGC)_n_ and (AG)_n_ in bread wheat *Triticum aestivum* var. Chinese Spring and its progenitors *T. urartu*, *A. speltoides*, *Ae. tauschii*, wild tetroploid emmer wheat *T. dicocoides*, and cultivated emmer wheat *T. dicoccum*. Results indicated that there are specific distribution patterns in different chromosomes from different species for each SSRs. They provided efficient visible markers for identification of some individual chromosomes and SSR sequence evolution tracing from the diploid progenitors to hexaploid wheat. During wheat speciation, the SSR sequence expansion occurred predominately in the centromeric and pericentromeric regions of B genome chromosomes accompanied by little expansion and elimination on other chromosomes. This result indicated that the B genome might be more sensitive to the “genome shock” and more changeable during wheat polyplodization.

**Conclusions:**

During the bread wheat evolution, SSRs including (AAC)_n_, (AAG)_n_, (AGC)_n_ and (AG)_n_ in B genome displayed the greatest changes (sequence expansion) especially in centromeric and pericentromeric regions during the polyploidization from *Ae. speltoides* S genome, the most likely donor of B genome. This work would enable a better understanding of the wheat genome formation and evolution and reinforce the viewpoint that B genome was originated from S genome.

**Supplementary Information:**

The online version contains supplementary material available at 10.1186/s12864-020-07364-6.

## Background

Polyploidization happens widely in angiosperms, and it has been estimated that more than 70% of flowering plants have experienced polyploidization in their evolutionary history [1]. Upon merging two or more different genomes into one nucleus, the nascent polyploid faces several challenges such as rescheduling chromosome pairing, gene expression and DNA replication, and reducing the cost of large genomes. To meet these challenges, the polyploid genome must undergo a series of genetic and epigenetic changes [2–7]. The epigenetic changes are mainly associated with methylation changes, while the genetic changes mainly involve a large number of sequence removal, genome rearrangements, rewiring of gene expression, and chromosome instability [4, 7–9]. All these events occurred immediately after polyploid formation, or on an evolutionary scale [7, 8, 10]. The polyploidization events might lead to the harmonious behaviour and activity of the different constituent genomes, and facilitated the establishment of the newly formed polyploids as successful species [1, 2, 6, 11]. Hence, polyploidization is an important source of stress that facilitates rapid genome evolution.

Bread wheat (*T. aestivum* L., 2n = 6x = 42, AABBDD) is a widely cultivated cereal crop composed of three distinct subgenomes and is believed to be a product of two large rounds of hybrid speciation (homoploid and polyploidy) [12, 13]. The first round of hybridization occurred between *T. urartu* (2n = 2x = 14, A^u^A^u^) and *Ae. speltoides* (a closely related source of the B genome, 2n = 2x = 14, SS) at approximately 0.5–0.36 million years ago, which formed the wild emmer wheat *T. turgidum* ssp. *dicocoides* (2n = 4x = 28, A^u^A^u^BB). The second hybridization happened between the domesticated emmer wheat *T. dicoccum* (2n = 4x = 28, A^u^A^u^BB) and *Ae. tauschii* (2n = 2x = 14, DD) at about 8000 years ago, which resulted in the emergence of the hexaploid bread wheat [13]. Following allopolyploidization of wheat, massive genomic rearrangements happened in response to this “genome shock”, which played an important role in wheat speciation and domestication [11]. These genomic changes were mainly involved in non-coding sequences, such as transposable elements and simple repetitive DNA sequences, which always underwent more extensive changes than low-copy sequences and coding sequences [4, 10]. However, how these genomic changes promoted wheat genome evolution still needs to be investigated. Moreover, as the B genome of wheat was more enriched in constitutive heterochromatic regions and more changeable [14–16], it was difficult to unequivocally identify the diploid donor species of B genome. Although extensive researches pointed that *Ae. speltoides* is likely the direct donor of all B genome chromosomes of wheat [16–18], more evidence is still needed.

Simple-sequence repeats (SSRs) belong to one class of repetitive sequences that are widely distributed throughout the wheat genomes, and some of them exist as large tandem arrays in the genome and can be localized on chromosomes using fluorescence in situ hybridization (FISH) [19–23]. Therefore, several SSRs have been widely used as cytological markers for chromosome identification and karyotype analysis. Their distribution patterns suggest that SSRs are mainly located in heterochromatic regions, especially the centromeric, pericentromeric and telomeric regions [21, 24, 25]. These regions could protect the repetitive sequences from the selective pressures, thus making it possible that satellite repeats could undergo significant modification during evolution, and may generate changes in their chromosomal location between closely related species, or different individuals [24], even among different generations of the same species. Thus, SSR sequences are the useful markers for tracing the genomic DNA sequence variations of chromosomal heterochromatic regions. Although the distribution of some SSRs on chromosomes was investigated in bread wheat and/or its progenitors [21, 23, 25–31], the distribution dynamics of SSRs on the chromosomes of bread wheat and its donors was not fully exploited due to the high polymorphism of SSR sequences and lack of comprehensive comparison of SSR distribution on chromosomes of bread wheat and its donors.

The main objective of this work was to analyze the distribution patterns of SSRs on chromosomes by FISH in wheat and its progenitors for tracing the possible genome changes during the process of wheat formation, especially changes that happened in the centromeric and pericentromeric regions. Investigating the genome evolution of bread wheat in the context of SSR distribution dynamics would enable us to have a better understanding of wheat genome origins and evolution.

## Results

### Screening of FISH positive SSR probes in bread wheat

**Fig. 1:**
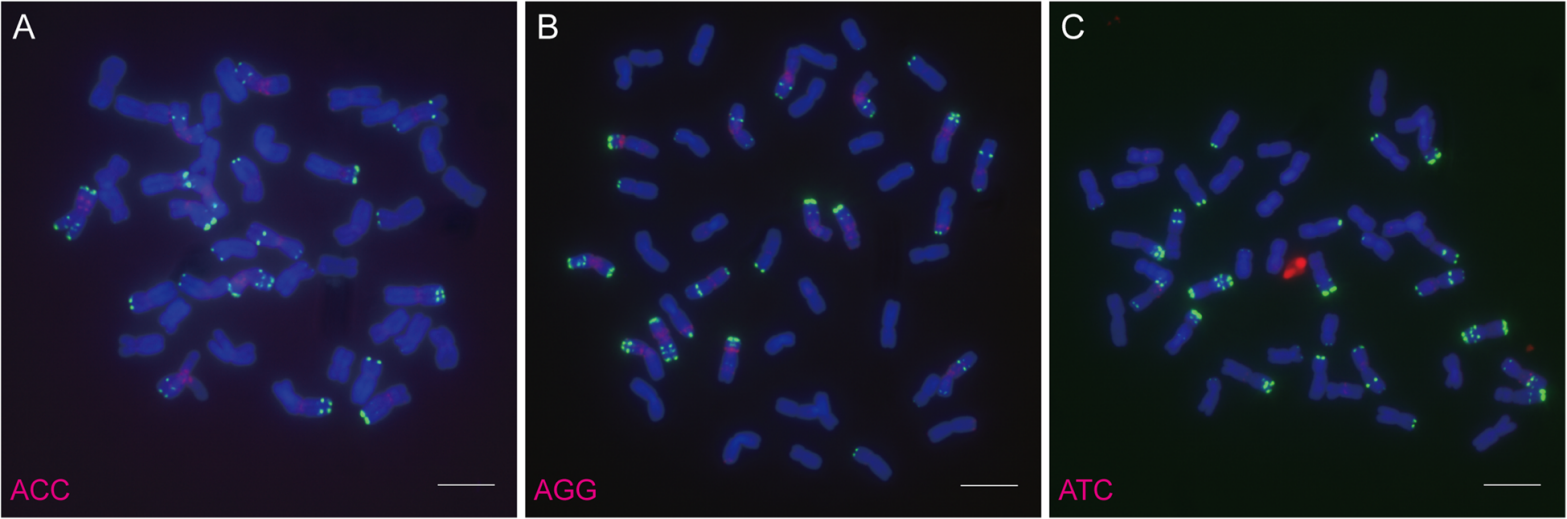
Distribution of (ACC)_n_, (AGG)_n_ and (ATC)_n_ on metaphase chromosomes of bread wheat *T. aestivum* var. Chinese Spring. **a**: (ACC)_n_ (red) and pSc119.2 (green); **b**: (AGG)_n_ (red) and pSc119.2 (green); **c**: (ATC)_n_ (red) and pSc119.2 (green). Bar = 10 μm

**Fig. 2:**
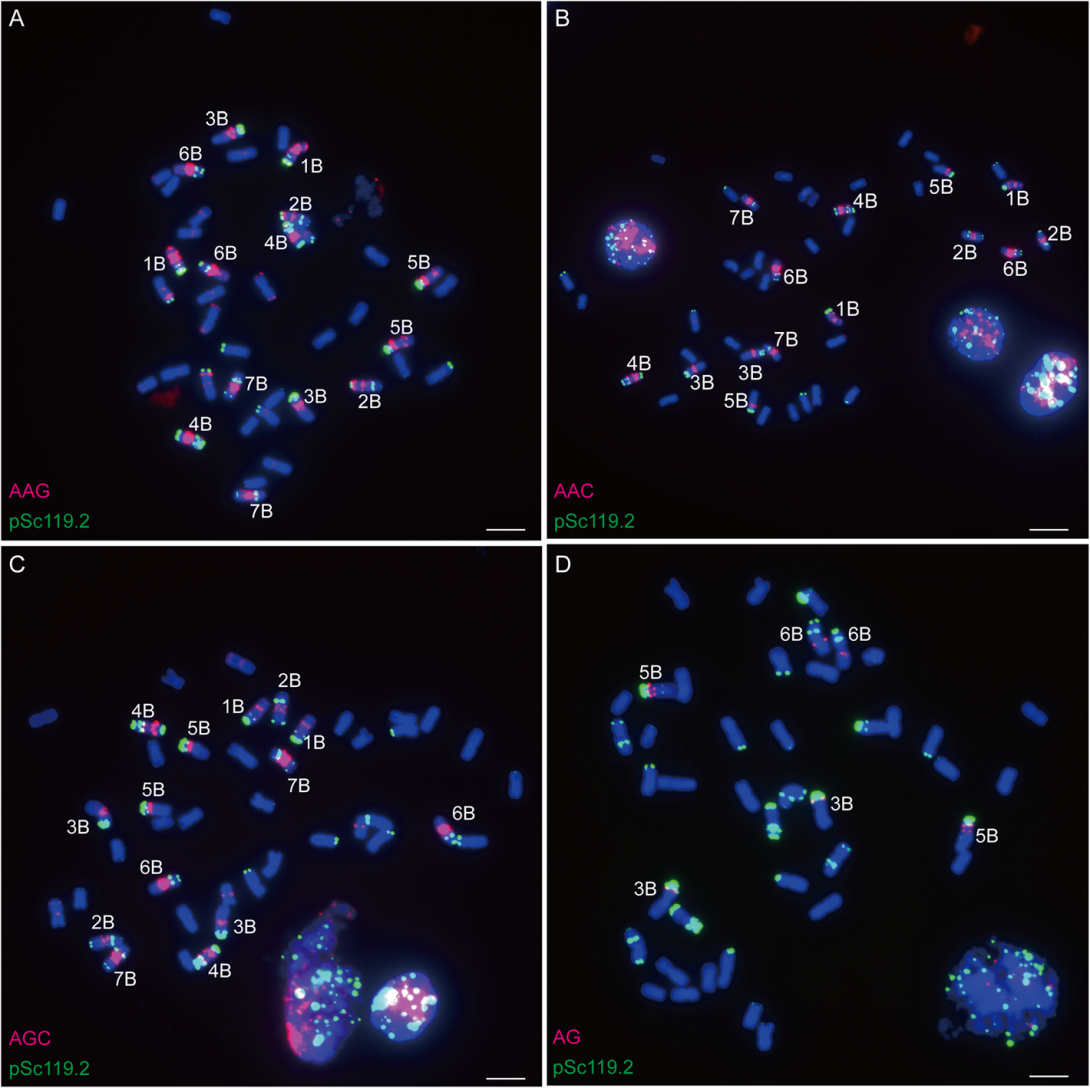
Distribution of (AAG)_n_, (AAC)_n_, (AGC)_n_ and (AG)_n_ on metaphase chromosomes of bread wheat *T. aestivum* var. Chinese Spring. **a**: (AAG)_n_ (red) and pSc119.2 (green); **b**: (AAC)_n_ (red) and pSc119.2 (green); **c**: (AGC)_n_ (red) and pSc119.2 (green); **d**: (AG)_n_ (red) and pSc119.2 (green). Bar = 10 μm

**Fig. 3:**
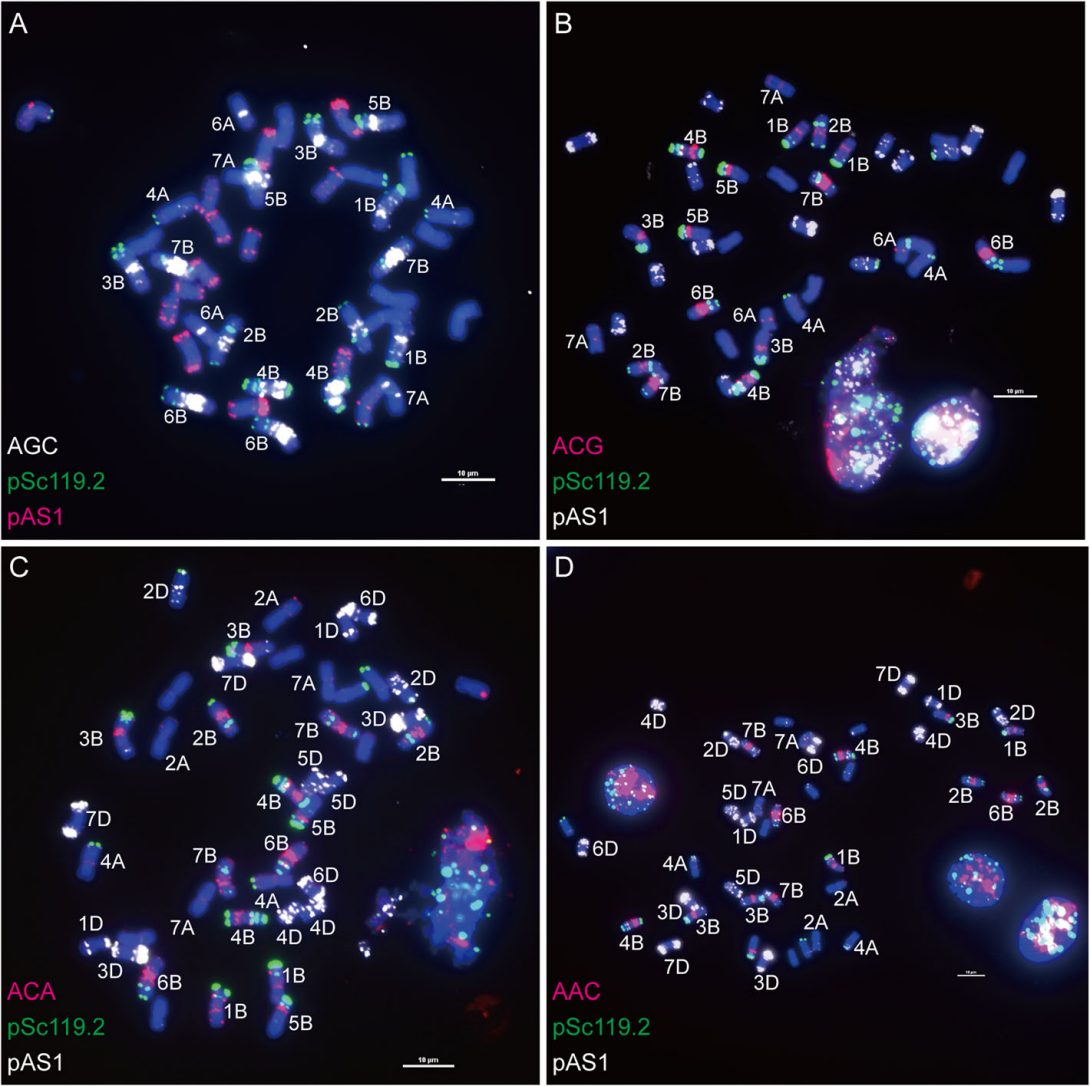
Distribution of (AGC)_n_, (ACG)_n,_ (ACA)_n_ and (AAC)_n_ on metaphase chromosomes of bread wheat *T. aestivum* var. Chinese Spring. **a**: (AGC)_n_ (white), pSc119.2 (green) and pAS1 (red); **b**: (ACG)_n_ (red), pSc119.2 (green) and pAS1 (white) probe; **c**: (ACA)_n_ (red), pSc119.2 (green) and pAS1 (white); **d**: (AAC)_n_ (red), pSc119.2 (green) and pAS1 (white). Bar = 10 μm

After analysis with Tandem Repeats Finder version 4.09 [32], 21 SSR motifs (Table 1) repeated more than 5 times in the wheat genome were labelled and used for FISH positive SSR probes screening. After hybridization with mitotic chromosomes of bread wheat using SSR sequences as the probes for FISH, a total of 6 SSR sequences, (AAC)_n_, (AAG)_n_, (ACA)_n_, (AG)_n_, (AGC)_n_ and (ACG)_n_, displayed strong and stable FISH signals and 3 SSR sequences, (AGG)_n_, (ATC)_n_ and (ACC)_n_ showed weak FISH signals on chromosomes of bread wheat (Figs. 1, 2 and 3). Among these 9 FISH positive SSR sequences, (ACA)_n_ and (AAC)_n_ showed similar signal distribution (Fig. 3a and b), and (AGC)_n_ and (ACG)_n_ showed similar signal distribution (Fig. 3c and d). (AAC)_n_, (AAG)_n_, (AGC)_n_ and (AG)_n_ were selected for extensive investigation.

**Table 1:** SSR primers for probe labeling

SSR motifs	Primers for probe labelling		FISH signals on wheat chromosomes
	Forward primer (5′-′3)	Reverse primer (5′-′3)	
AAC	AACAACAACAACAACAACAACAACAACAAC	GTTGTTGTTGTTGTTGTTGTTGTTGTTGTT	Strong
AAG	AAGAAGAAGAAGAAGAAGAAGAAGAAGAAG	CTTCTTCTTCTTCTTCTTCTTCTTCTTCTT	Strong
AGC	AGCAGCAGCAGCAGCAGCAGCAGCAGCAGC	GCTGCTGCTGCTGCTGCTGCTGCTGCTGCT	Strong
ACG	ACGACGACGACGACGACGACGACGACGACG	CGTCGTCGTCGTCGTCGTCGTCGTCGTCGT	Strong
ACA	ACAACAACAACAACAACAACAACAACAACA	TGTTGTTGTTGTTGTTGTTGTTGTTGTTGT	Strong
AG	AGAGAGAGAGAGAGAGAGAG	CTCTCTCTCTCTCTCTCTCT	Strong
AGG	AGGAGGAGGAGGAGGAGGAGGAGGAGGAGG	CCTCCTCCTCCTCCTCCTCCTCCTCCTCCT	Weak
ATC	ATCATCATCATCATCATCATCATCATCATC	GATGATGATGATGATGATGATGATGATGAT	Weak
ACC	ACCACCACCACCACCACCACCACCACCACC	GGTGGTGGTGGTGGTGGTGGTGGTGGTGGT	Weak
GT	GTGTGTGTGTGTGTGTGTGT	ACACACACACACACACACAC	None
TA	TATATATATATATATATATA	TATATATATATATATATATA	None
GC	GCGCGCGCGCGCGCGCGCGC	GCGCGCGCGCGCGCGCGCGC	None
CGG	CGGCGGCGGCGGCGGCGGCGGCGGCGGCGG	CCGCCGCCGCCGCCGCCGCCGCCGCCGCCG	None
GTC	GTCGTCGTCGTCGTCGTCGTCGTCGTCGTC	GACGACGACGACGACGACGACGACGACGAC	None
GGA	GGAGGAGGAGGAGGAGGAGGAGGAGGAGGA	CCTCCTCCTCCTCCTCCTCCTCCTCCTCCT	None
TCC	TTCTTCTTCTTCTTCTTCTTCTTCTTCTTC	GAAGAAGAAGAAGAAGAAGAAGAAGAAGAA	None
GCG	GCGGCGGCGGCGGCGGCGGCGGCGGCGGCG	CGCCGCCGCCGCCGCCGCCGCCGCCGCCGC	None
TCA	TCATCATCATCATCATCATCATCATCATCA	TGATGATGATGATGATGATGATGATGATGA	None
GAG	GAGGAGGAGGAGGAGGAGGAGGAGGAGGAG	CTCCTCCTCCTCCTCCTCCTCCTCCTCCTC	None
GCAC	GCACGCACGCACGCACGCACGCACGCACGCACGCACGCAC	GTGCGTGCGTCGTGCGTGCGTGCGTGCGTGCGTGCGTGC	None
TAAT	TAATTAATTAATTAATTAATTAATTAATTAATTAATTAAT	ATTAATTAATTAATTAATTAATTAATTAATTAATTAATTA	None

### The distribution patterns of (AAC)_n_ on chromosomes of bread wheat and its progenitors

**Fig. 4:**
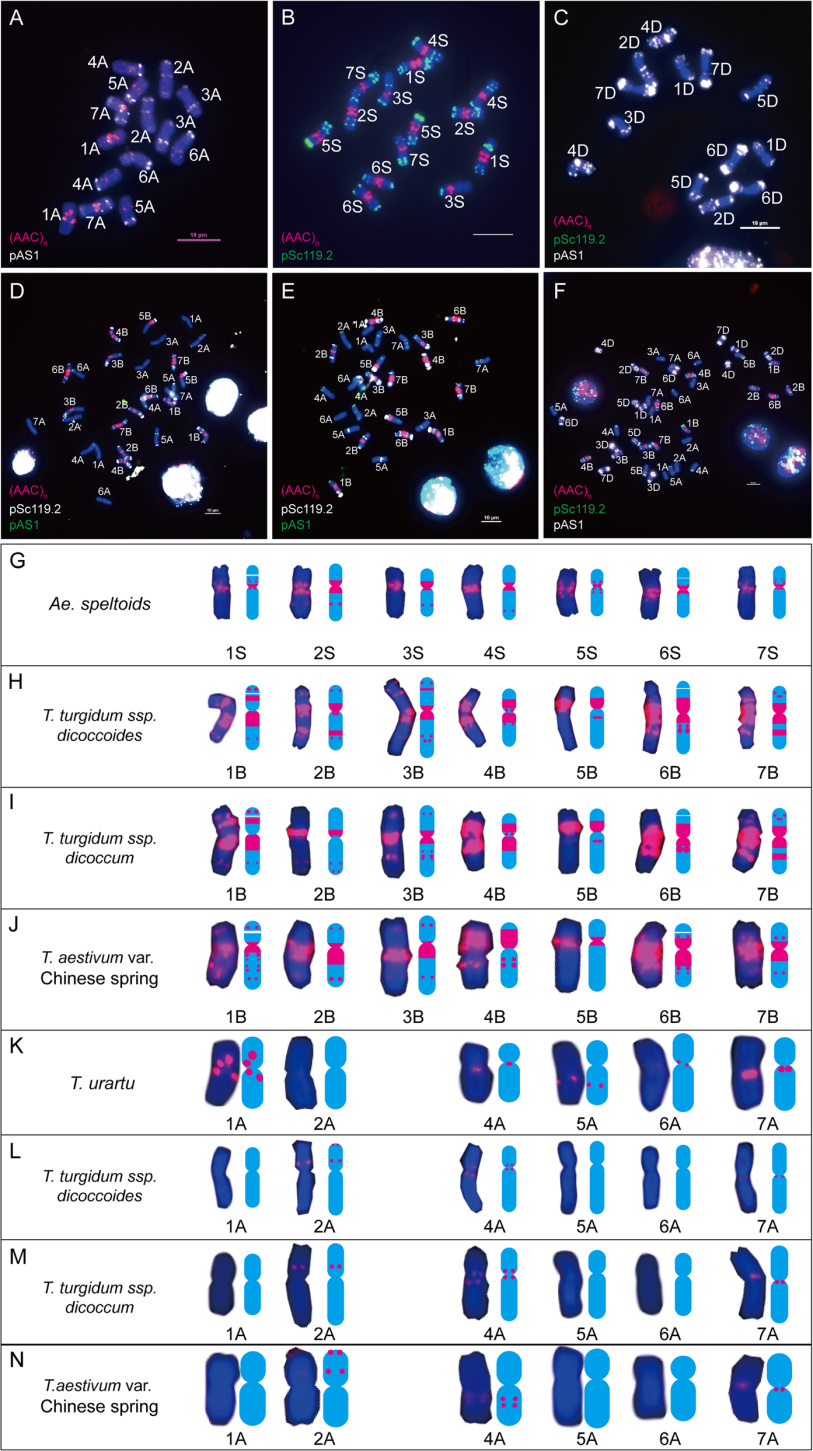
(AAC)_n_ distribution on chromosomes of wheat and its progenitors. **a**: *T. urartu*; **b**: *Ae. speltoides*; **c**: *Ae. tauschii*; **d**: *T. turgidum* ssp. *dicoccoides*; **e**: *T. turgidum* ssp*. dicoccum*; **f**: *T. aestivum* var. Chinese Spring; **g**: Images and mode patterns of genome S individual chromosomes taken from *Ae. speltoides* (**b**); **h**: Images and mode patterns of genome B individual chromosomes taken from *T. turgidum* ssp. *dicoccoides* (**d**); **i**: Images and mode patterns of genome B individual chromosomes taken from *T. turgidum* ssp. *dicoccum* (**e**); **j**: Images and mode patterns of genome B individual chromosomes taken from *T. aestivum* var. Chinese Spring (**f**); **k**: Images and mode patterns of genome A individual chromosomes taken from *T. urartu* (**a**); **l**: Images and mode patterns of genome A individual chromosomes taken from *T. turgidum* ssp. *dicoccoides* (**d**); **m**: Images and mode patterns of genome A individual chromosomes taken from *T. turgidum* ssp. *dicoccum* (**e**); **n**: Images and mode patterns of genome A individual chromosomes taken from *T. aestivum* var. Chinese Spring (**f**). To highlight the red signals, other colours of signals were removed artificially in **g** - **j**. Red signals, (AAC)_n_. Bar = 10 μm

In Chinese Spring, (AAC)_n_ displayed a strong clustered hybridization pattern on all chromosomes of the B genome, and some of the adjacent signals tended to be coalesced and appeared as a condensed large band (Fig. 4f and j). Sporadically weak signals were detected on chromosomes 2A, 4A and 7A (Fig. 4f and n). No obvious signals were observed on D genome chromosomes (Fig. 4f). In *T. urartu*, the (AAC)_n_ motif produced several strong spot-like signals in the pericentromeric regions of both arms of chromosome 1A, the centromeric region of chromosome 4A, a signal on proximal third part of long arm of chromosome 5A, a weak signal in the centromeric region of chromosome 6A and a strong signal in the pericentromeric region of the long arm of chromosome 7A (Fig. 4a and k). In *Ae. tauschii*, (AAC)_n_ only produced weaker spot-like signals in centromeric regions of chromosome 4D (Fig. 4c). In *Ae. speltoides*, strong signals were observed in the centromeric and pericentromeric regions of all the chromosomes (S genome), which are weaker and fewer than those on chromosomes of the B genome in Chinese Spring (Fig. 4b and g). In wild emmer wheat (*T. turgidum* ssp. *dicoccoides*), the wild relative of durum wheat, strong condensed broad band signals were observed on both arms of chromosomes 1B, 2B, 4B, 6B and 7B, short arms of chromosome 5B and pericentromeric regions of chromosome 3B; in addition to band-like signals, dot-like signals could also be observed on both arms of chromosomes 1B, 2B, 3B, 4B and 4A, on long arms of chromosomes 5B, 6B and 7A, and on short arms of 7B and 2A chromosomes (Fig. 4d, h and l). In domesticated emmer wheat (*T. turgidum* ssp. *dicoccum*), similar signal distribution patterns to those of wild emmer wheat were observed, except chromosomes 2B and 2A, which showed narrower band signals and fewer dot-like signals (Fig. 4e, i and m).

Comparing the chromosomal distribution patterns of (AAC)_n_ between bread wheat and its diploid progenitors*,* obvious (AAC)_n_ sequence expansion was observed on chromosomes 1B, 2B, 7B and 2A; sequence elimination was detected on chromosomes 1A, 5A, 6A and 4D; and both expansion and elimination were observed on chromosomes 2B, 3B, 6B, 4A and 7A in bread wheat (Fig. 4). However, the distribution changes of (AAC)_n_ from progenitors to the bread wheat genome was mainly the sequence expansion in the B genome (especially in chromosomes 1B, 2B, 4B, 6B and 7B). Comparing the chromosomal distribution patterns of (AAC)_n_ between bread wheat and its tetraploid progenitors*,* sequence elimination was observed on 1B, 3B, 4B, 5B and 7B chromosomes; whereas, both sequence expansion and elimination were detected on 2B chromosomes in bread wheat (Fig. 4, Additional files 1 and 2).

### The distribution patterns of (AAG)_n_ on chromosomes of bread wheat and its progenitors

**Fig. 5:**
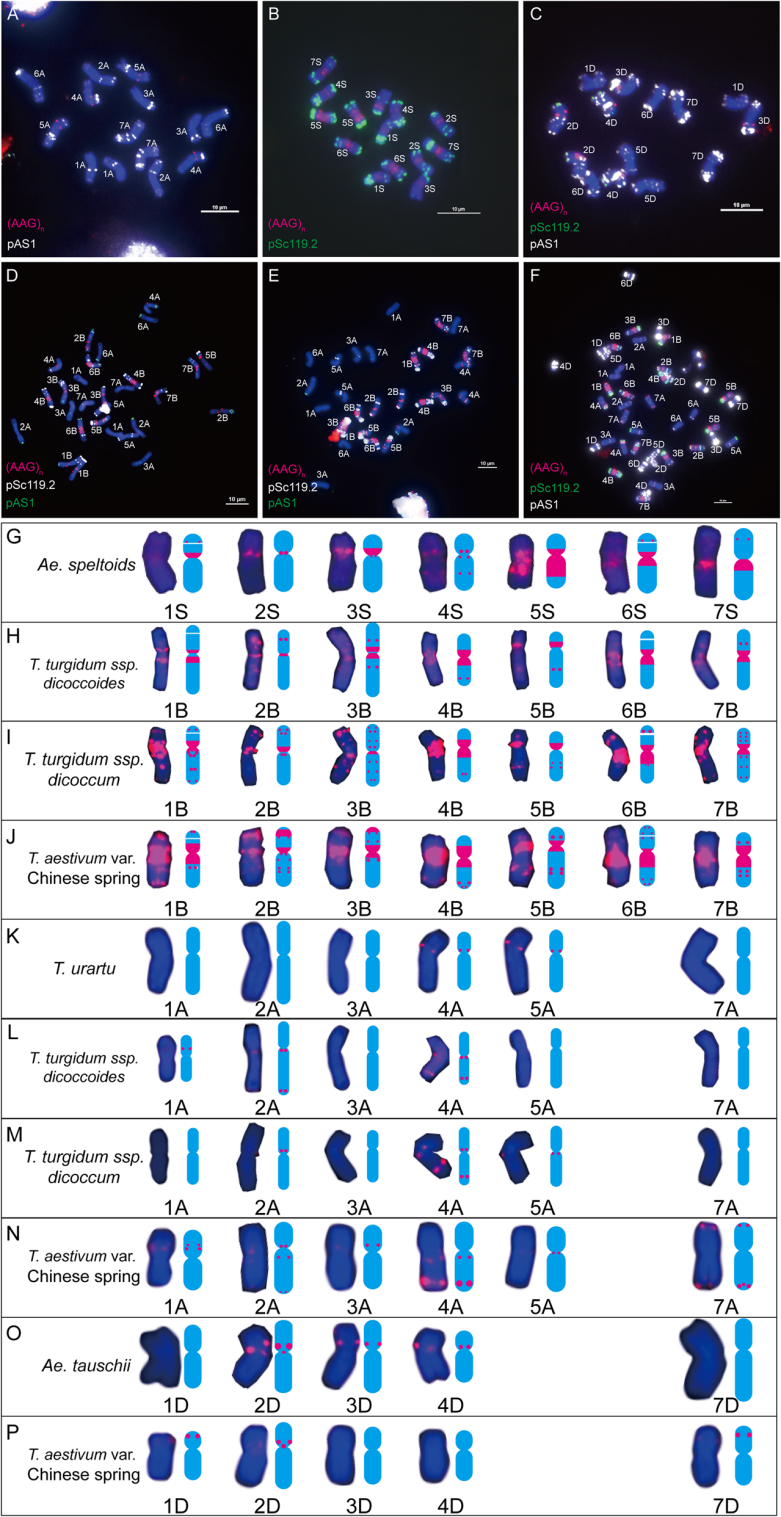
(AAG)_n_ distribution on chromosomes of wheat and its progenitors. **a**: *T. urartu*; **b**: *Ae. speltoides*; **c**: *Ae. tauschii*; **d**: *T. turgidum* ssp. *dicoccoides*; **e**: *T. turgidum* ssp. *dicoccum*; **f**: *T. aestivum* var. Chinese Spring; **g**: Images and mode patterns of genome S individual chromosomes taken from *Ae. speltoides* (**b**); **h**: Images and mode patterns of genome B individual chromosomes taken from *T. turgidum* ssp. *dicoccoides* (**d**); **i**: Images and mode patterns of genome B individual chromosomes taken from *T. turgidum* ssp. *dicoccum* (**e**); **j**: Images and mode patterns of genome B individual chromosomes taken from *T. aestivum* var. Chinese Spring (**f**); **k**: Images and mode patterns of genome A individual chromosomes taken from *T. urartu* (**a**); **l**:Images and mode patterns of genome A individual chromosomes taken from met *T. turgidum* ssp. *dicoccoides* (**d**); **m**: Images and mode patterns of genome A individual chromosomes taken from *T. turgidum* ssp. *dicoccum* (**e**); **n**: Images and mode patterns of genome A individual chromosomes taken from *T. aestivum* var. Chinese Spring (**f**); **o**: Images and mode patterns of genome D individual chromosomes taken from *Ae. tauschii* (**c**); **p**: Images and mode patterns of genome D individual chromosomes taken from *T. aestivum* var. Chinese Spring (**f**). To highlight the red signals, other colours of signals were removed artificially in **g** - **j**. Red signals, (AAG)_n_. Bar = 10 μm

In Chinese Spring, (AAG)_n_ showed strong signal distribution in centromeric and pericentromeric regions of all B genome chromosomes. Compared with the distribution of (AAC)_n_, (AAG)_n_ were more distally located. In addition, unlike (AAC)_n_, obvious subtelomeric signals of (AAG)_n_ were present on chromosomes 1B, 2B and 3B, and a strong intercalary signal band could also be found on 5BS (Fig. 5f and j). There were sporadically weak (AAG)_n_ signals in pericentromeric regions of chromosomes 2A, 3A, and 4A and the centromeric region of chromosome 5A and interstitial region of the chromosome 1A arm; apparent signals in the subtelomeric regions of chromosome 4A; and obvious telomeric signals on chromosome 7A (Fig. 5f and n). (AAG)_n_ showed weak signals in subtelomeric regions of chromosomes 1D and 7D, and pericentromeric regions of chromosome 2D (Fig. 5f and p).

In *T. urartu*, the (AAG)_n_ motif produced two dot-like signals in the pericentromeric regions of 4A and 5A (Fig. 5a and k). In *Ae. speltoides*, the FISH signals of (AAG)_n_ were observed in the centromeric and pericentromeric regions of all the chromosomes, and they were slightly weaker than those of (AAC)_n_, except chromosome 5S (Fig. 5b and g), while most signals were weaker than those in Chinese Spring. In *Ae. tauschii*, (AAG)_n_ displayed signals in the pericentromeric regions of chromosomes 2D, 3D and 4D (Fig. 5c and o). In wild and emmer wheats, strong condensed band-like signals were observed in the pericentromeric regions of all chromosomes; dot-like signals could be detected on both arms of chromosome 3B, short arms of chromosomes 2B and 7B, and long arms of chromosomes 3B, 4B, 5B, 2A and 4A (Fig. 5d, h and l). In domesticated emmer wheat, broader band-like signals than wild emmer wheat were observed in the pericentromeric regions of all chromosomes; more dot-like signals than those of wild emmer wheat were observed on both arms of chromosomes 1B, 2B, 3B and 7B, short arm of chromosome 6B, and long arms of chromosomes 4B, 5B, 2A and 4A (Fig. 5e, i and m).

Similar to (AAC)_n_, (AAG)_n_ was mainly dispersed on B genome chromosomes. The main changes in (AAG)_n_ distribution from diploid progenitors to the bread wheat genome were the sequence expansions on chromosomes 1B, 2B, 3B, 4B, 6B, 7B and 4A and the sequence elimination of chromosomes 5B, 2D, 3D and 4D during wheat formation. The main changes of (AAG)_n_ distribution from tetraploid progenitors to the bread wheat genome were the sequence expansions on all B and A genome chromosomes (Fig. 5, Additional files 1 and 2).

### The distribution patterns of the (AGC)_n_ motif in bread wheat and its progenitors

**Fig. 6:**
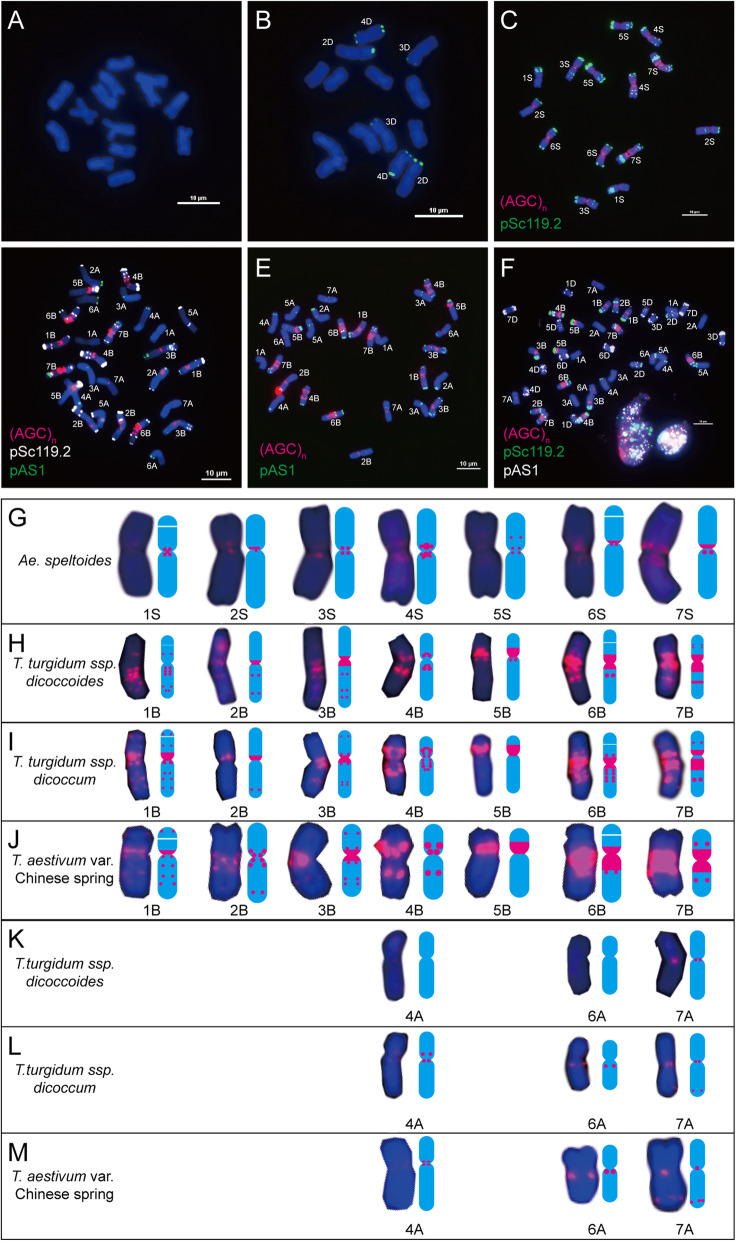
(AGC)_n_ distribution on chromosomes of wheat and its progenitors. **a**: *T. urartu*; **b**: *Ae. speltoides*; **c**: *Ae. tauschii*; **d**: *T. turgidum* ssp. *dicoccoides*; **e**: *T. turgidum* ssp. *dicoccum*; **f**: *T. aestivum* var. Chinese Spring; **g**: Images and mode patterns of genome S individual chromosomes taken from *Ae. speltoides* (**b**); **h**: Images and mode patterns of genome B individual chromosomes taken from *T. turgidum* ssp. *dicoccoides* (**d**); **i**: Images and mode patterns of genome B individual chromosomes taken from *T. turgidum* ssp. *dicoccum* (**e**); **j**: Images and mode patterns of genome B individual chromosomes taken from *T. aestivum* var. Chinese Spring (**f**); **k**: Images and mode patterns of genome A individual chromosomes taken from *T. turgidum* ssp. *dicoccoides* (**d**); **l**: Images and mode patterns of genome A individual chromosomes taken from *T. turgidum* ssp. *dicoccum* (**e**); **m**: Images and mode patterns of genome A individual chromosomes taken from *T. aestivum* var. Chinese Spring (**f**). To highlight the red signals, other colours of signals were removed artificially in **g** - **j**. Red signals, (AGC)_n_. Bar = 10 μm

In Chinese Spring, the FISH pattern of (AGC)_n_ on B genome chromosomes was more similar to (AAC)_n_, but with lower signal density, especially on chromosomes 1B and 2B (Fig. 6f and j); dot-like signals were detected in the pericentromeric regions of chromosomes 4A, 6A and 7A, and subtelomeric regions of chromosome 7A (Fig. 6f and j), and no signals were detectable on D genome chromosomes (Fig. 6f). In *Ae. speltoides*, similar to the distribution patterns of (AAC)_n_ and (AAG)_n_, the (AGC)_n_ motif signals were also concentrated in the pericentromeric regions, but with lower intensity (Fig. 6c and g). No (AGC)_n_ signals were detectable in the diploid species *T. urartu* and *Ae. tauschii* (Fig. 6a and b). In wild emmer wheat, band-like signals were observed on the pericentromeric regions of chromosomes 2B, 3B, 5B, 6B and 7B; dot-like signals could be detected on both arms of chromosomes 1B and 4B, long arms of chromosomes 2B, 3B and 6B, short arms of chromosome 7B, and pericentromeric regions of chromosomes 4B and 7A (Fig. 6d, h and k). In domesticated emmer wheat, band-like signals were observed on the pericentromeric regions of all B genome chromosomes, except chromosome 4B; dot-like signals were observed on both arms of chromosome 1B, 3B, and 7B, short arms of chromosome 4A, long arms of chromosomes 2B, 6B, 6A and 7A, and pericentromeric regions of chromosomes 4B, 6B, 4A and 7A (Fig. 6e, i and l).

By comparison of the chromosomal distribution patterns of (AGC)_n_ between bread wheat and its progenitors, obvious sequence expansion of (AGC)_n_ was observed on chromosomes 1B-7B (especially 4B, 6B and 7B), 4A, 6A and 7A; both expansion and elimination of (AGC)_n_ sequences were observed on chromosomes on 4B and 5B of bread wheat (Fig. 6, Additional files 1 and 2). In the progenitors of bread wheat, similar to (AAC)_n_ and (AAG)_n_, (AGC)_n_ FISH signals were predominantly detected on B genome chromosomes and rarely detected on A and D genome chromosomes. The expansion of (AGC)_n_ along B genome chromosomes was also the main change during wheat formation.

### The distribution patterns of the (AG)_n_ motif in bread wheat and its progenitors

**Fig. 7:**
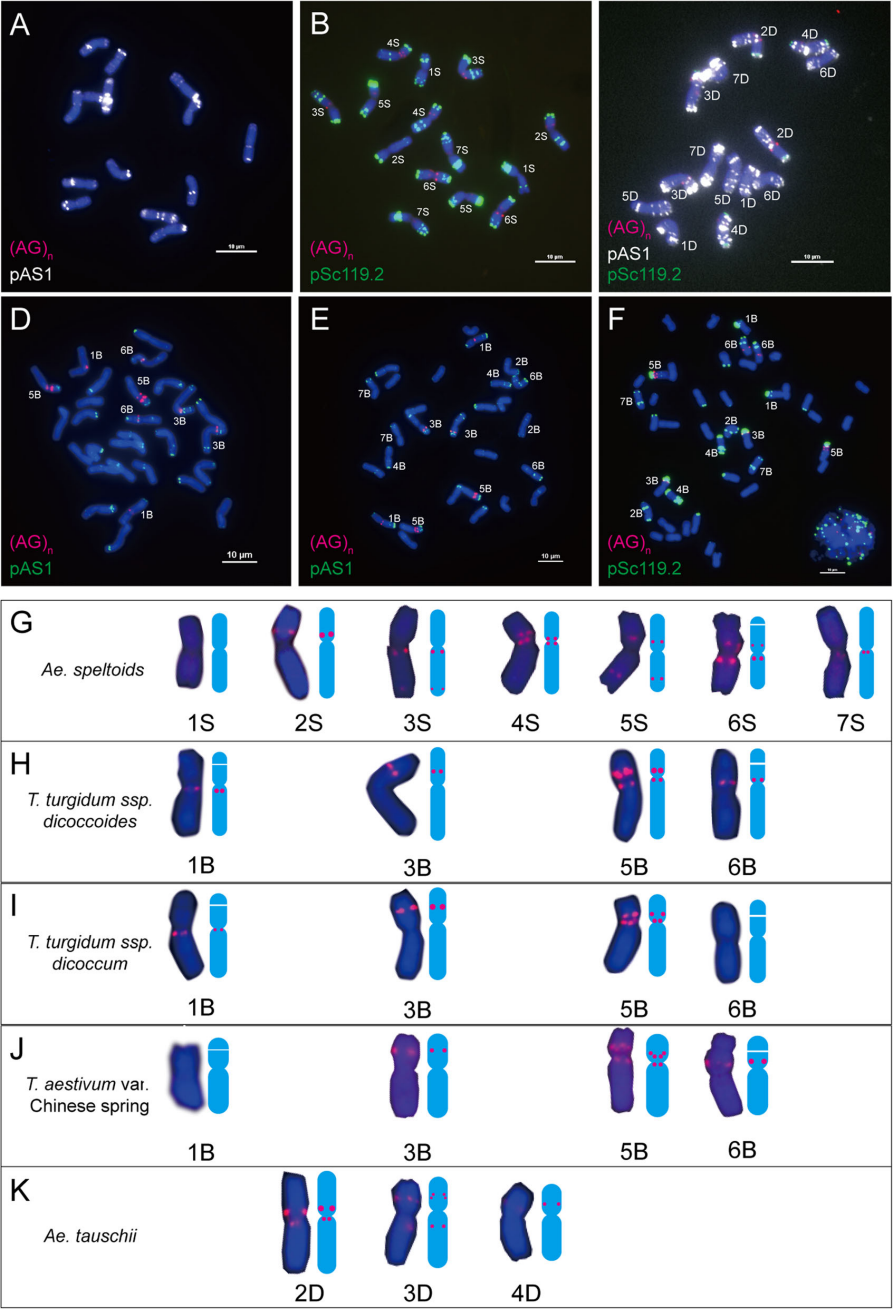
(AG)_n_ distribution on chromosomes of wheat and its progenitors. **a**: *T. urartu*; **b**: *Ae. speltoides*; **c**: *Ae. tauschii*; **d**: *T. turgidum* ssp*. dicoccoides*; **e**: *T. turgidum* ssp*. dicoccum*; **f**: *T. aestivum* var. Chinese Spring; **g**: Images and mode patterns of genome S individual chromosomes taken from *Ae. speltoides* (**b**); **h**: images and mode patterns of genome B individual chromosomes taken from *T. turgidum* ssp. *dicoccoides* (**d**); **i**: Images and mode patterns of genome B individual chromosomes taken from *T. turgidum* ssp. *dicoccum* (**e**); **j**:Images and mode patterns of genome B individual chromosomes taken from *T. aestivum* var. Chinese Spring (**f**); **k**: Images and mode patterns of genome D individual chromosomes *Ae. tauschii* (**c**). To highlight the red signals, other colours of signals were removed artificially in **g** - **j**. Red signals, (AG)_n_. Bar = 10 μm

Different from other SSRs, (AG)_n_ signals were only clustered in the pericentromeric regions of chromosomes 3B, 5B and 6B in Chinese Spring (Fig. 7f and j), and no detectable signals were observed on the other chromosomes. In *Ae. speltoides*, (AG)_n_ signals were observed in the pericentromeric or near the pericentromeric regions of chromosomes 2S to 7S, and the distal regions of long arms of chromosomes 3S and 5S, but there was no detectable signal on chromosome 1S (Fig. 7b and g); in addition, spot-like signals were observed near the pericentromeric regions of chromosomes 2D, 3D and 4D in *Ae. tauschii* (Fig. 7c and k), even though no D genome chromosome located signals were observed in Chinese Spring (Fig. 7f and j). In wild and emmer wheat, spot-like signals were observed on the pericentromeric regions of chromosomes 1B, 5B and 6B, and short arms of chromosome 3B (Fig. 7d and h). In domesticated emmer wheat, a similar signal distribution pattern to those of wild emmer wheat was observed except on chromosome 6B (Fig. 7e and i).

In the comparison of the chromosomal distribution patterns of (AG)_n_ between bread wheat and its progenitors, (AG)_n_ sequence elimination was observed on chromosomes 2B, 4B, 6B, 7B, 2D, 3D and 4D in bread wheat; and both expansion and elimination were observed on 3B, 5B, and 6B chromosomes of wheat (Fig. 7, Additional files 1 and 2). The elimination of the (AG)_n_ sequence in the B genome was the main changes during wheat formation.

## Discussion

Bread wheat, an allohexaploid cereal crop, possesses a very large genome with over 80% of repetitive sequences [14, 19]. Even though the whole genome sequence data of bread wheat and its two diploid progenitors (*Ae. tauschii* and *T. urartu*) are publicly available [14, 33–35], the accurate sequencing, assembly, and chromosomal localization of repetitive sequences remains challenging, especially for the unsequenced B genome donor. Moreover, the relationship between the S genome of *Ae. speltoides* and the B genome of durum and bread wheats is still being debated, even though *Ae. speltoides* is considered the most likely B donor [15, 36]. SSRs, which are abundant components present in wheat, is an efficient tool to study genomic alterations in eukaryotes [6, 17, 18, 20, 22, 30]. In this study, the distribution dynamics of SSRs on chromosomes of bread wheat and its progenitors were comprehensively investigated by FISH.

Short stretches of SSRs (20 ~ 250 bp) are normally undetectable by FISH, and the FISH detectable regions should be the regions enriched in long stretches of SSR sequences. In this study, 21 SSR sequences were labeled (Table 1) and hybridized with the chromosomes of bread wheat Chinese Spring, and only 6 SSR sequences, (AAC)_n_, (AAG)_n_, (ACA)_n_, (AG)_n_, (AGC)_n_ and (ACG)_n_, displayed strong and stable FISH signals (Figs. 1, 2 and 3). This result suggested that these 6 SSRs were prone to forming long stretches of sequences in the wheat genome and could produce detectable FISH signals on chromosomes of bread wheat.

The selected SSRs, (AAC)_n_, (AAG)_n_, (AG)_n_ and (ACG)_n_ had already been used as FISH probes to study genetic diversity or genomic reconstruction either in wheat, wheat relatives or wheat progenitor species [17, 21, 23, 25–27, 29–31, 33, 37–40], but had not been comprehensively compared among wheat and its diploid and tetraploid progenitors. In this study, a systematic study was performed to investigate the distribution patterns of different SSRs on the chromosomes of wheat and its diploid and tetraploid progenitors, obvious SSR signal changes were observed from diploid donor to hexaploid wheat, which support the previous studies that the wheat genome has underwent extensive changes during its polyploidization and evolution [16, 36]. In addition, stronger and wider FISH signals than previous studies were detected in this study, which might be caused by the difference of probe labeling method. Using a PCR method in the absence of template, rather than random-primer labeling method or direct synthesized method [21–23, 25, 39], could produce longer probes and might reflect the actual location of large SSR clusters.

SSR sequences were not randomly distributed in wheat genomes, as their distribution on chromosomes depending on the motif, chromosome and genome, as demonstrated by Cuadrado et al. [21]. Although the size of SSR repeat units in wheat still needs to be confirmed, large SSR clusters could be located and compared in this study. In consistence with the studies that constitutive heterochromatic regions and SSRs are more abundant in the B genome chromosomes than those of A and D genomes [14, 36, 41, 42], most signals of (AAC)_n_, (AAG)_n_, (ACG)_n_ and (AG)_n_ were located on the B genome chromosome, followed by A genome chromosomes, and were least likely to be found on D genome chromosomes, and each SSR has its unique distribution patterns on different genomes and different chromosomes (Figs. 4, 5, 6 and 7). The results suggested that there would be a high heterogeneity of SSRs in wheat genome, especially B genome.

To integrate our SSR FISH results with their physical position in the genomes, the physical position of (AAC)_n_, (AAG)_n_, (AGC)_n_, and (AG)_n_ were predicted using the web server B2DSC (http://mcgb.uestc.edu.cn/b2dsc) (Additional files 3, 4, 5 and 6) [27]. The prediction results indicated that high copy number SSR tandem repeats prone to cluster on chromosomes of wheat, especially the centromeric and pericentromeric regions of B genome chromosomes. Low copy number SSR repeats were more likely dispersed in the genomes of *Ae. tauschii* and *T. urartu*, which were not long enough to be detected by FISH, especially for the (AG)_n_ sequence (Additional files 3, 4, 5 and 6). As the *Ae. speltoides* has not been sequenced yet, the physical location of SSR sequences in the genome was not analyzed here. As expected, the distribution of large SSR clusters in the predicted physical map was consistent with the results of FISH analysis, but the SSR sequences mapped by FISH were more concentrated, which showed the longer and abundant SSR sequence locations. In addition, more SSR repeats prone to form small clusters and widely distributed in genome, especially in genomes of *Ae. tauschii* and *T. urartu*. These small SSR clusters were not long enough to be detected by FISH (Additional files 3, 4, 5 and 6).

Following polyploidization, the wheat genome has undergone massive genomic rearrangements, including chromosome variation, sequence amplification and elimination [7, 11–13, 16]. To trace its genome evolution dynamics during its formation and evolution, karyotypes of wheat and its diploid and tetraploid progenitors based on our four SSR FISH results were constructed, and differences were analysed in terms of the abundance and localization of SSR motifs between different genomes and chromosomes. During wheat formation, compared with its progenitors, more SSR FISH signal changes were detected as SSR sequence expansion and/or elimination on B genome chromosomes (judged by the rough strength and number of FISH signals). Briefly and importantly, during wheat formation, the main distribution changes of our studied four SSRs from diploid progenitors to the bread wheat genome should be the sequence expansion on chromosomes 1B, 2B, 4B, 6B and 7B for (AAC)_n_; the sequence expansion on chromosomes 1B, 2B, 3B, 4B, 6B, 7B and 4A and the sequence elimination on chromosomes 5B, 2D, 3D and 4D for (AAG)_n_; the sequence expansion on chromosomes 1B-7B (especially 4B, 6B and 7B), 4A, 6A and 7A and both expansion and elimination on chromosomes 4B and 5B for (AGC)_n_ and the sequence elimination on chromosomes 2B, 4B, 6B, 7B, 2D, 3D and 4D; and both expansion and elimination on chromosomes 3B, 5B, and 6B for (AG)_n_. These results suggested that SSR sequences preferred to move and/or amplify around their original locations rather than different genomes or chromosomes during wheat formation. This phenomenon could be explained because variability of SSRs in the genome is primarily the result of slipped-strand mispairing followed by replication and recombination, or repair errors, which could change the lengths of microsatellites [43]. Among the four SSR probes, signal changes of (AAG)_n_ was the biggest across the B genome chromosomes, followed by (AAC)_n_, (ACG)_n_ and (AG)_n_ (Figs. 4, 5, 6 and 7), which suggested that different SSRs evolved at different speed across the B genome chromosomes. For (AAC)_n_, signal changes on 1B, 4B, 6B and 7B were larger than those on 2B, 3B and 5B; For (AAG)_n_, signal changes on 1B, 4B, 5B and 7B were larger than those on 2B, 3B and 6B; For (ACG)_n_, signal changes on 3B, 4B, 5B, 6B and 7B were larger than those on 1B and 2B. These results is consistent with the view that genome differentiation during wheat allopolyploidization from S to B proceeds at different speeds over the chromosomes, which was revealed by genome-wide exon sequencing and resultant phylogenetic analysis [36].

The origin of the B genome of wheat had been debated by researchers for a very long time, as it evolved “at a higher rate of evolution” than the A and D genome, the B genome of wheat is now more different from S genome of *Ae. speltoides* [15, 36], which was also elucidated in this study. But even so, most SSR FISH signals on chromosomes of *Ae. speltoides* could be found in chromosomes of the bread wheat (Figs. 4, 5, 6 and 7), which supports the viewpoint that *Ae. speltoides* is likely to be the direct donor of all B genome chromosomes of wheat [15, 36]. Moreover, more sequence changes were detected between wheat and its diploid progenitors rather than between wheat and its tetraploid progenitors, and the result suggested that the genome shock brought by the first hybridization event might be larger than the second hybridization event during wheat formation.

Revealed by phylogenetic analysis and FISH, centromeric satellites in wheat genome have undergone rapid changes in the three subgenomes and satellite signals decreased from diploid to hexaploid wheat [44]. In this study, it is obvious that the SSR sequence expansion occurred predominately in the centromeric and pericentromeric regions of B genome chromosomes during wheat formation, despite the SSR copy number variation in the centromeric region was not precisely calculated. These results suggested that the wheat centromeric and pericentromeric regions were sensitive to “genome shock” and evolved rapidly during the evolution of wheat. All of these findings support the idea that the wheat genome is a dynamic system with a high level of plasticity [44], and a changing sequence repertoire shaped by sequence losses and expansion.

Our SSR FISH results indicated that the genome of wheat has evolved substantially following its polyploidization, and the rearrangement of SSRs might be important for facilitating wheat genome evolution and stabilizing chromosomes of different subgenomes. To address this, more work needs to be performed, such as the investigation of newly formed wheat species or relatives by more SSR FISH. Moreover, new studies might uncover the underlying mechanisms responsible for the widescale genome rearrangement in polyploids.

## Conclusion

We investigated the genome evolution by comprehensively comparing the distribution patterns of (AAC)_n_, (AAG)_n_, (AGC)_n_ and (AG)_n_ with FISH in bread wheat and its progenitors *T. urartu*, *Ae. speltoides, Ae. tauschii, T. dicocoides and T. dicoccum*. Obvious SSR signal changes were observed from diploid donor to hexaploid wheat in this study, especially SSR sequence expansion happened in the pericentromeric and centromeric regions of B genome chromosomes. The results suggested SSRs were efficient tools for tracing wheat genome polyploidization and evolution. The B genome of wheat, especially the centromeric and pericentromeric regions of which, might be more sensitive to “genome shock” and evolved rapidly during the course of bread wheat formation, which further demonstrated the viewpoint that the B genome of bread wheat was more enriched in SSR sequences and evolved “at a higher rate of evolution” than the A and D genome [15, 36].

## Methods

### Materials

Plant materials used in this study included *T. urartu* (2n = 2x = 14, A^u^A^u^), *Ae. speltoides* (2n = 2x = 14, SS), *Ae. tauschii* (2n = 2x = 14, DD), wild tetraploid emmer wheat *T. dicocoides* (2n = 4x =28, A^u^A^u^BB), cultivated emmer wheat *T. dicoccum* (2n = 4x = 28, A^u^A^u^BB), and *T. aestivum* var. Chinese Spring (2n = (2n = 6x = 42, AABBDD). The source of all the materials have been listed in Additional file 7.

### The screening of FISH positive SSR probes

Bread wheat Chinese Spring was used to screen FISH positive SSR probes. Before performing FISH, the whole genome sequence of wheat cv. Chinese Spring (IWGSC RefSeqv1.0) was downloaded from URGI (https://wheat-urgi.versailles.inra.fr/Seq-Reposi tory/Assemblies) and analysed using *Tandem* Repeats Finder version 4.09 with default parameters (http://tandem.bu.edu/trf/trf.basic.submit.html) [32]. SSR motifs repeated more than 5 times were chosen to hybridize with mitotic chromosomes of bread wheat.

### The preparation of SSRs probes

SSR sequences were prepared using a PCR method in the absence of template, which is similar to the preparation of the telomere-specific probes [45]. Primers used to prepare the SSR probes were designed using primer 5.0 software and synthesized by the Shanghai Sangon Biological Engineering Technology Engineering Service Co., Ltd. and they are listed in Table 1. Briefly, SSRs sequences were prepared by PCRs that were carried out in a 100 μL PCR system: 10 μL of 10× PCR buffer, 2 units of Taq polymerase (Takara Bio, Shiga, Japan), 200 μM for each dNTP and 200 nM for each primer. Amplification consisted of 10 cycles each consisting of 1 min at 94 °C, 30 s at 55 °C, and 1 min at 72 °C, followed by 30 cycles each consisting of 1 min at 94 °C, 30 s at 60 °C, 90 s at 72 °C, and one final step of 5 min at 72 °C. Smear DNA bands should be detected using 1.4% agarose gel electrophoresis; and if not, then a second round of PCR was performed under the same conditions, using 3 μL of PCR products from the first round of PCR as template.

The probe labelling was conducted as previously described earlier [38]. The labelling system included 10 μL of PCR product described above, 2 μL of nick translation buffer, 2 μL of dNTP (−dCTP) mix, 0.5 μL of Texas Red-5-dCTP (1 mM), 0.5 μL of DNase I (100 mU/μL), and 5 μL of DNA polymerase I (10 U/μL).

The repetitive sequence pSc119.2, originally isolated from *Secale cereale* by McIntyre et al. [37], and pAs1, originally isolated from *Ae. tauschii* by Nagaki et al. [46], were labelled to identifying the B and D genome chromosomes of wheat, respectively.

### Chromosome preparation and FISH

Seeds were germinated on moist filter paper at 25 °C, and root tips of 1–2 cm in length were excised and treated with N_2_O for 2 h at room temperature before fixing in ethanol-acetic acid (3:1). Chromosome spread preparation and FISH were performed as previously described earlier [38]. Briefly, root tips were digested at 37 °C for 1 h in an enzyme solution of 0.5% of pectolyase Y-23 (Kikkoman Co., Tokyo, Japan) and 1% cellulose Onozuka R-10 (Yakult Honsha Co., Ltd. Minato-ku, Tokyo, Japan), and dropped onto the slides. Air-dried slides were cross linked by exposure to UV light, then denatured at 100 °C for 5 min with 6 μL of diluted probe mix on the spreads. After hybridization overnight at 55 °C, the slides were washed with 2× SSC and counterstained with DAPI. Slides were examined using a Nikon Ni-E fluorescence microscope and photographed with a Nikon DS-Ri2 camera system (Nikon CEE GmbH, Wien, Austria).

### Physical mapping of SSR sequences

To predict the physical location of SSR sequences, the SSR motifs (20 repeat units) were blasted against the genomes of wheat, *T. urartu* and *Ae. tauschii* respectively using B2DSC (http://mcgb.uestc.edu.cn/b2dsc) with default parameters [27].

## Supplementary Information

**Additional file 1.** SSR sequence dynamics observed between wheat and its diploid progenitors.

**Additional file 2.** SSR sequence dynamics observed between wheat and its tetroploid progenitors.

**Additional file 3.** Physical mapping of (AAC)_n_ on chromosomes of wheat and its diploid progenitors by using the web server B2DSC (http://mcgb.uestc.edu.cn/b2dsc) with default parameters for the blast and filter steps [27]. Yellow bars, the distribution of Oligo-CCS1 corresponding to the positions of centromeres of wheat. Blue-to-red bars, the number of HSPs per Mbp of SSR sequences (20 repeat units).

**Additional file 4.** Physical mapping of (AAG)_n_ on chromosomes of wheat and its diploid progenitors by B2DSC, using default parameters for the blast and filter steps. Yellow bars, the distribution of Oligo-CCS1 corresponding to the positions of centromeres of wheat. Blue-to-red bars, the number of HSPs per Mbp of SSR sequences (20 repeat units).

**Additional file 5.** Physical mapping of (AGC)_n_ on chromosomes of wheat and its diploid progenitors by B2DSC, using default parameters for the blast and filter steps. Yellow bars, the distribution of Oligo-CCS1 corresponding to the positions of centromeres of wheat. Blue-to-red bars, the number of HSPs per Mbp of SSR sequences (20 repeat units).

**Additional file 6.** Physical mapping of (AG)_n_ on chromosomes of wheat and its diploid progenitors by B2DSC, using default parameters for the blast and filter steps. Yellow bars, the distribution of Oligo-CCS1 corresponding to the positions of centromeres of wheat. Blue-to-red bars, the number of HSPs per Mbp of SSR sequences (20 repeat units).

**Additional file 7.** The accession list of wheat and its progenitor materials in this study.

## Data Availability

The datasets analysed during the current study are available in the repository of European Bioinformatics Institute (EMBL-EBI, https://www.ebi.ac.uk/), with accession number GCA_900519105. All the other data, including SSR primers used for probe labeling, the summary of the SSR sequence dynamics detected by FISH, the physical position of SSR sequences predicted using bioinformatics method and the source of all species studied here, can be found in the article itself and its supplementary data.

## Abbreviations

SSR: Simple sequence repeat
FISH: Fluorescence in situ hybridization
PCR: Polymerase chain reaction
DAPI: 4′,6-diamidino-2-phenylindole
