# Supplementary figures and images for: Genome evolution during bread wheat formation unveiled by the distribution dynamics of SSR sequences on chromosomes using FISH
Source: BMC Genomics. 2021 Jan 14;22:55. doi: 10.1186/s12864-020-07364-6 (PMC7809806; doi:10.1186/s12864-020-07364-6)

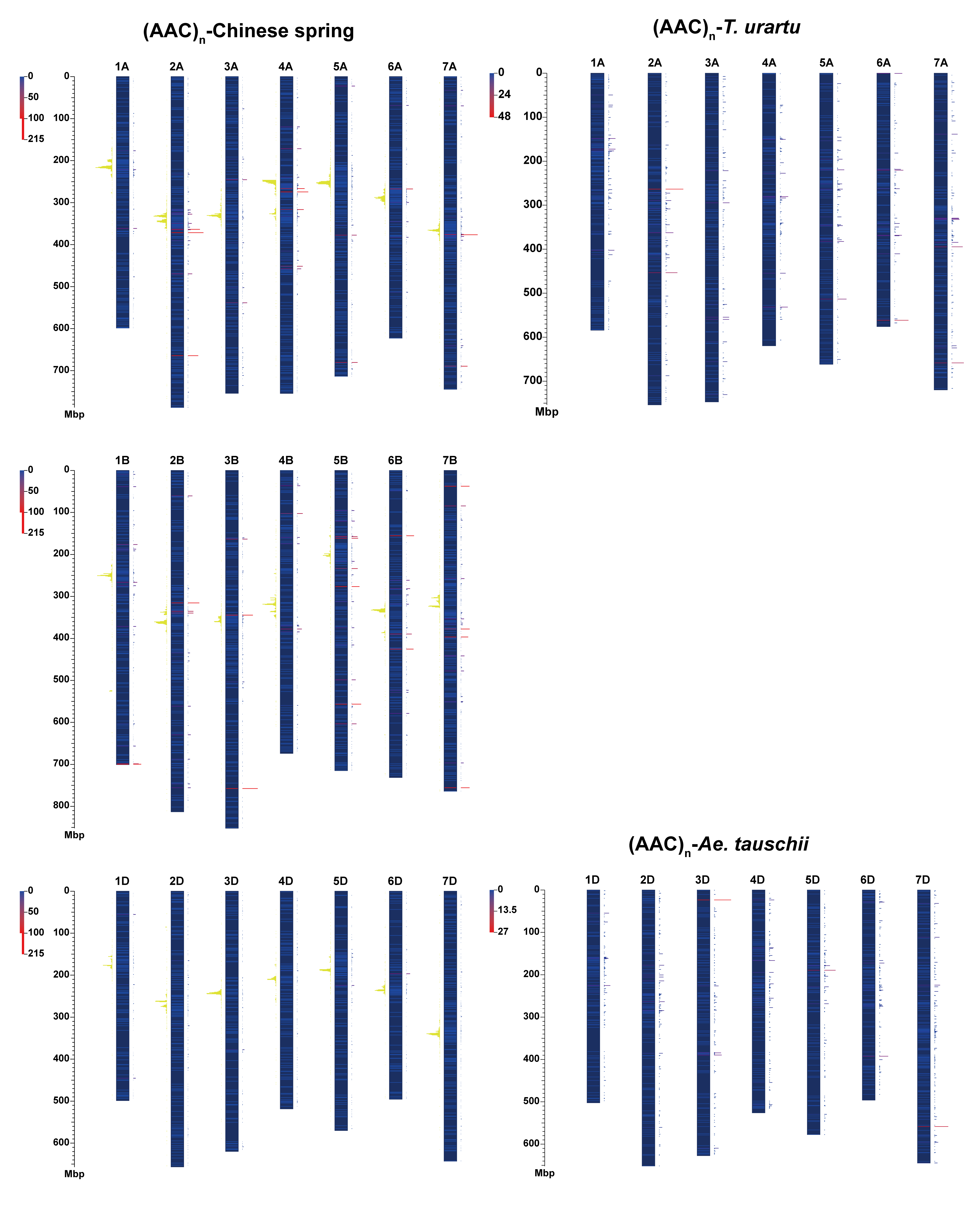

Supplement: Supplementary file 3 — Additional file 3. Physical mapping of (AAC)n on chromosomes of wheat and its diploid progenitors by using the web server B2DSC (http://mcgb.uestc.edu.cn/b2dsc) with default parameters for the blast and filter steps [27]. Yellow bars, the distribution of Oligo-CCS1 corresponding to the positions of centromeres of wheat. Blue-to-red bars, the number of HSPs per Mbp of SSR sequences (20 repeat units). [file 12864_2020_7364_MOESM3_ESM.tif]

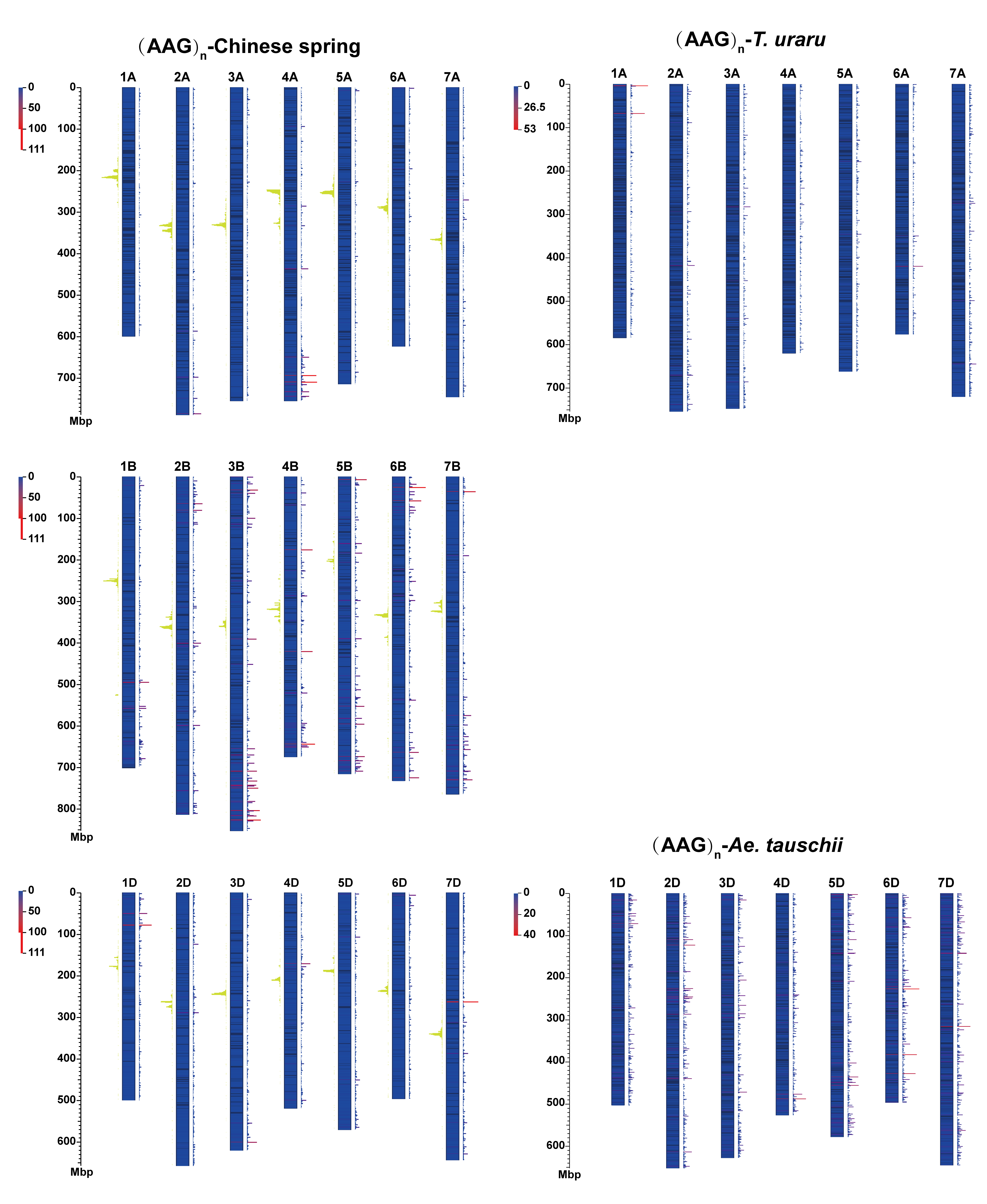

Supplement: Supplementary file 4 — Additional file 4. Physical mapping of (AAG)n on chromosomes of wheat and its diploid progenitors by B2DSC, using default parameters for the blast and filter steps. Yellow bars, the distribution of Oligo-CCS1 corresponding to the positions of centromeres of wheat. Blue-to-red bars, the number of HSPs per Mbp of SSR sequences (20 repeat units). [file 12864_2020_7364_MOESM4_ESM.tif]

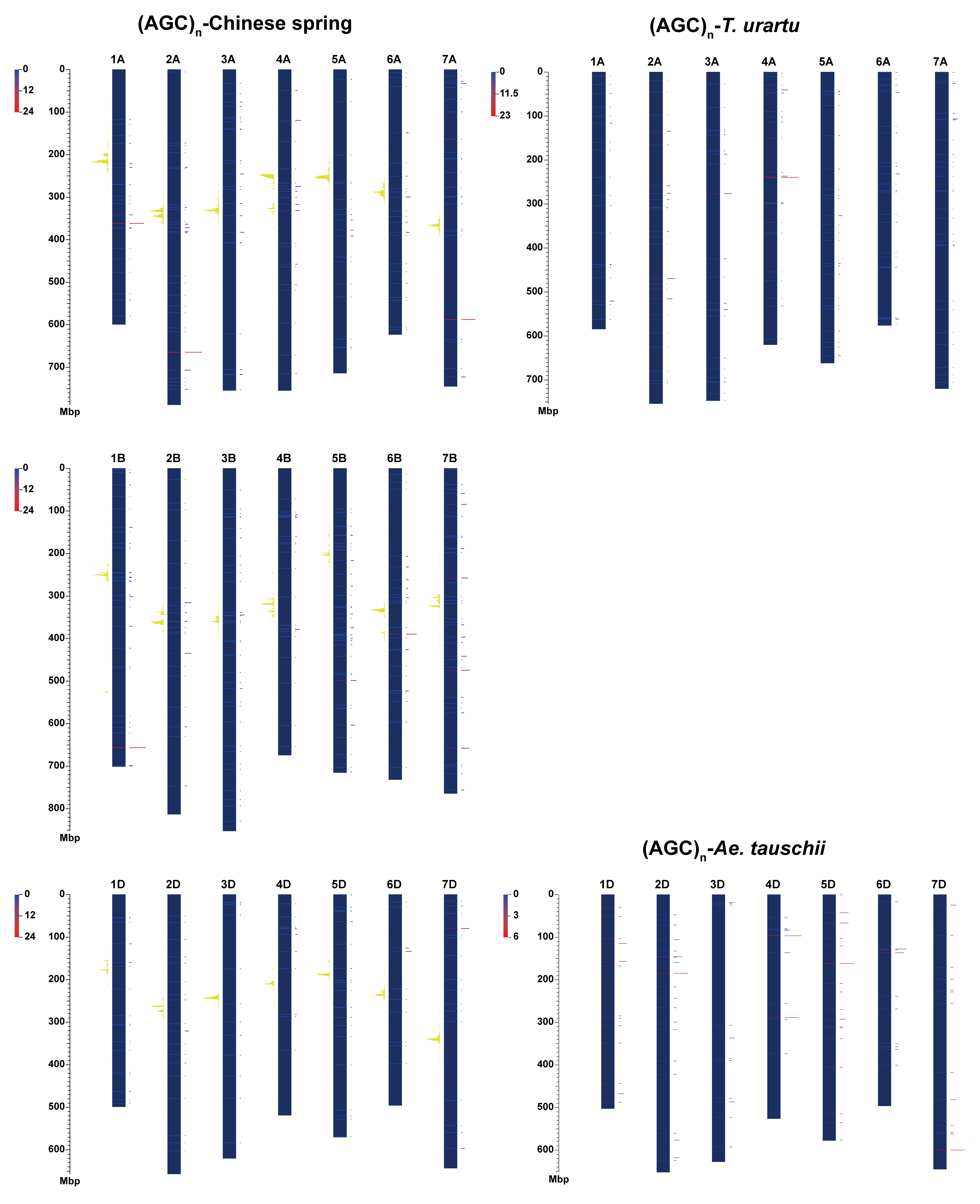

Supplement: Supplementary file 5 — Additional file 5. Physical mapping of (AGC)n on chromosomes of wheat and its diploid progenitors by B2DSC, using default parameters for the blast and filter steps. Yellow bars, the distribution of Oligo-CCS1 corresponding to the positions of centromeres of wheat. Blue-to-red bars, the number of HSPs per Mbp of SSR sequences (20 repeat units). [file 12864_2020_7364_MOESM5_ESM.tif]

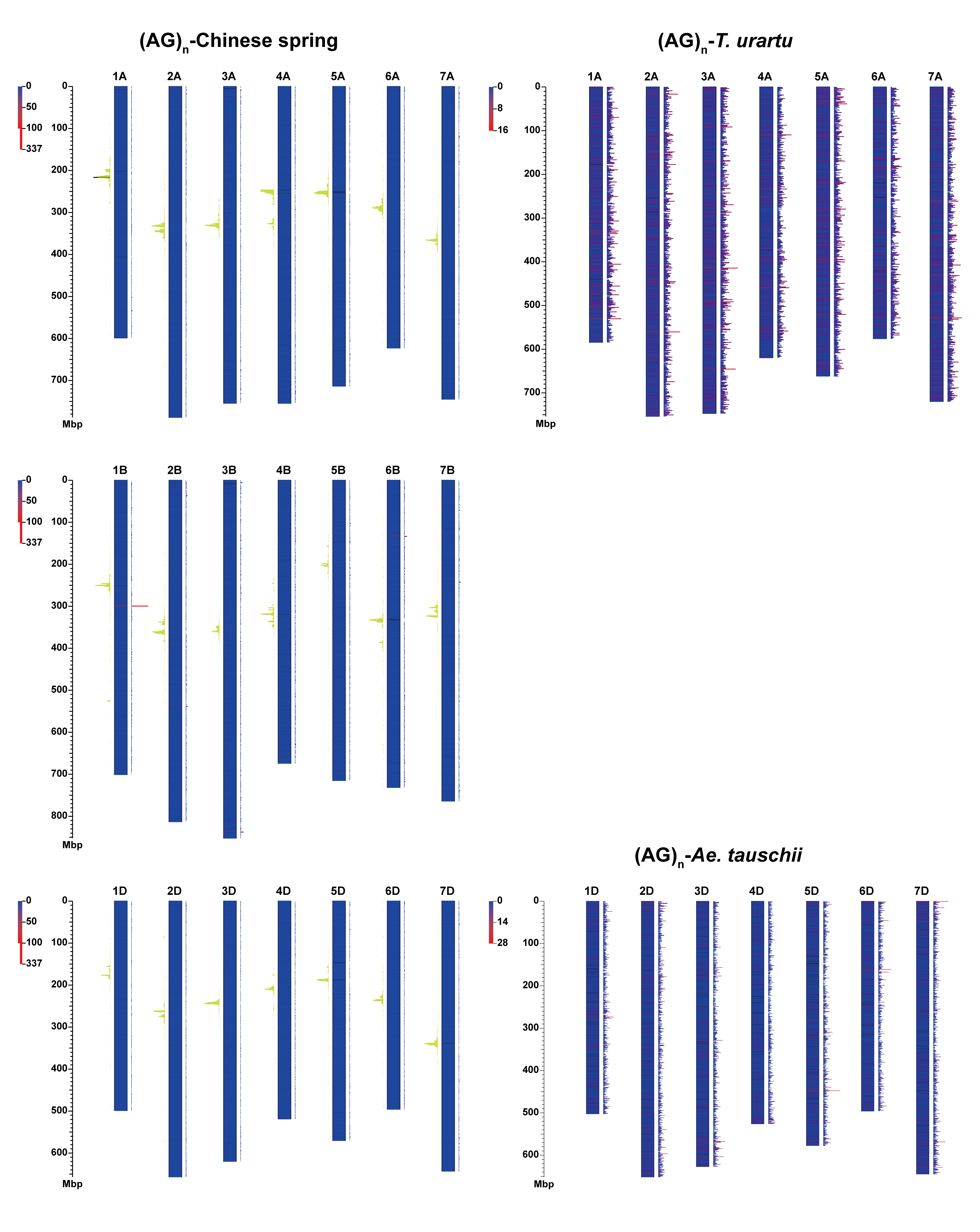

Supplement: Supplementary file 6 — Additional file 6. Physical mapping of (AG)n on chromosomes of wheat and its diploid progenitors by B2DSC, using default parameters for the blast and filter steps. Yellow bars, the distribution of Oligo-CCS1 corresponding to the positions of centromeres of wheat. Blue-to-red bars, the number of HSPs per Mbp of SSR sequences (20 repeat units). [file 12864_2020_7364_MOESM6_ESM.tif]
